# The Relationship Between Playing Formations, Team Ranking, and Physical Performance in the Serie A Soccer League

**DOI:** 10.3390/sports12110286

**Published:** 2024-10-22

**Authors:** Cristian Savoia, Francesco Laterza, Antonio Lucadamo, Vincenzo Manzi, Vito Azzone, Samuel A. Pullinger, Catherine E. Beattie, Maurizio Bertollo, Dario Pompa

**Affiliations:** 1 The Research Institute for Sport and Exercise Sciences, The Tom Reilly Building, Liverpool John Moores University, Liverpool L3 5AH, UK; cristiansavoia@gmail.com; 2 Department of Wellbeing, Nutrition and Sport, Pegaso Open University, 80143 Naples, Italy; francesco.laterza@univr.it (F.L.); vincenzo.manzi@unipegaso.it (V.M.); 3 Department of Neurosciences, Biomedicine and Movement Sciences, University of Verona, 37129 Verona, Italy; 4 Department of Law, Economics, Management and Quantitative Methods (DEMM), University of Sannio, 82100 Benevento, Italy; alucadam@unisannio.it; 5 Italian Football Federation, 00198 Rome, Italy; vitoazzone79@gmail.com; 6 Sport Science Department, Inspire Institute of Sport, Karnataka 583123, India; pullinger.s@hotmail.com; 7 School of Allied Health Professions, Keele University, Newcastle ST5 5BG, UK; catherinebeattie3@gmail.com; 8 Sport Science & Medical Department, Wrexham Association Football Club, Wrexham LL11 2AH, UK; 9 Behavioral Imaging and Neural Dynamics (BIND) Center, Department of Medicine and Aging Sciences, University “G. d’Annunzio” of Chieti-Pescara, 66013 Chieti, Italy; dario.pompa@unich.it

**Keywords:** soccer, running performance, playing formation, team ranking

## Abstract

The influence of playing formations and team ranking on the physical performance of professional soccer players is an open question that needs to be explored. The present study aimed to investigate the impact of these factors on the physical exertion of Serie A soccer players. We analyzed match data from 375 players, categorizing teams based on their final ranking and comparing performance across different playing formations. The Kruskal–Wallis test and the Dunn test with Bonferroni adjustment revealed that high-ranking (HR) teams exhibited a higher percentage of high-intensity (HI) accelerations compared to mid-ranking teams, suggesting the critical role of HI efforts in achieving favorable match outcomes. Moreover, the 4-3-3 playing formation was associated with greater acceleration demands than other formations, particularly in HR teams. Our study also established benchmarks for various performance metrics, enabling coaches to assess player performance and identify potential signs of overtraining. These findings contribute to a deeper understanding of the physical demands in soccer and offer practical implications for coaches and players in optimizing training and performance strategies.

## 1. Introduction

The execution of soccer-related bouts requires a large physiological load on players during competition. Research has indicated that their activity profile is role-positioning dependent [1,2,3,4,5,6], and that contextual variables, such as the playing formation [7,8] and the ranking of the opponents [9], can significantly affect the locomotor activity of professional soccer players. Although some research has explored the influence of these variables on locomotor activity, as Plakias and Michailidis [9] pointed out, the findings remain contradictory. In particular, certain studies reported that team quality does not significantly impact running performance [10], whereas others reported the opposite [11]. The same was true when the opponents’ level was considered. Modric et al. [10] found no significant effect of the opponents’ level on locomotor activity, while Gonçalves et al. [12,13] observed that facing strong opponents increases the total distance covered by a team. Morgans et al. [7] found that not only does the playing formation affect players’ physical performance, but also that the playing style (e.g., defensive, direct, possession-based) plays a role in the resultant locomotor activity of professional soccer players. However, Bradley et al. [14] reported that high- and very high-intensity running distances (e.g., running over 20 km/h) were similar in 4-4-2, 4-3-3, and 4-5-1 playing formations when ball possession was not considered. These contradictory results indicate the need to investigate this topic in more depth to better understand what the training should be focused on.

When analyzing the locomotor activity of soccer players, it is crucial to recognize the association between high-intensity activities and the most decisive soccer game events [15]. Consequently, high-intensity actions warrant careful consideration in this analysis.

One of these high-intensity activities is represented by high-intensity running, which is a crucial element of soccer performance. Moreover, it serves as a valuable indicator of physical performance in soccer [6], differentiating various levels of play [6,16], the tactical role of players [17,18], and fluctuations throughout the competitive season [6]. It is even sensitive to physiological changes associated with the end of a training program [19].

The traditional speed-category approach, neglecting acceleration and deceleration, provides only a partial understanding of the actual game’s physiological and external load experienced during a match [20,21]. By considering the energy expenditure estimated from acceleration, deceleration, and speed following the method proposed by Osgnach et al. [20], a more comprehensive description of match demands has become possible. Osgnach’s method [20] quantifies players’ activity as the distance covered within arbitrarily chosen energy-expenditure categories, referred to as metabolic power (MP).

Greig and Siegler [22] highlighted the importance of sprinting and acceleration in contributing to muscular fatigue due to their high neuromuscular demand. However, using absolute acceleration thresholds can lead to misclassification of high-intensity acceleration events, underestimating those with high initial running speed and overestimating those with low initial speeds [23].

With the method proposed by Sonderegger et al. [24], it is possible to consider the initial running speed and the population-specific maximal acceleration values at various initial speeds, thus improving the accuracy of detecting high-intensity acceleration actions.

Video match analysis is a valuable tool for evaluating soccer players’ performance. This technique, initially introduced and used to monitor the work-rate profiles of elite players [17,25], has become indispensable for assessing physical and tactical behavior in training and competition. It enables complex analytical evaluations of a large sample size. In fact, a multiple-camera video system is pivotal in the analysis of high-intensity bouts, where detailed information can be collected [3].

Given the contradictory findings in previous research, as highlighted by Plakias and Michailidis [9] in their analysis of Turkish first division soccer data, and given the literature gap in this research field in the context of the Italian Championship, this exploratory study aims to investigate how ranking and playing formations influence the physical exertion of professional soccer players in the Italian First Division (Serie A). The secondary aim of this study is to compile data from professional soccer players in different roles and playing formations to provide benchmarks to facilitate the interpretation of players’ performance, as well as to detect symptoms of overtraining, thus offering valuable insights for coaches to provide better training prescriptions.

## 2. Materials and Methods

### 2.1. Sample

Using semi-automatic tracking, we analyzed 212 professional soccer players from 20 Italian Serie A teams during the 2018/2019 season, comprising 48 attackers (27.2 ± 2.8 years, height 184.2 ± 4.7 cm and body mass 79.5 ± 6.9 kg), 41 box-to-box midfielders (26.4 ± 2.5 years, height 179.3 ± 5.1 cm and body mass 76.4 ± 5.7 kg), 52 central defenders (28.7 ± 3.0 years, height 183.2 ± 5.4 cm and body mass 80.3 ± 6.8 kg), 12 central midfielders (25.7 ± 2.6 years, height 175.3 ± 4.8 cm and body mass 74.5 ± 6.2 kg), 26 wide defenders (26.2 ± 2.9 years, height 178.4 ± 5.2 cm and body mass 76.5 ± 5.2 kg), and 33 wide midfielders (26.8 ± 2.7 years, height 177.2 ± 4.5 cm and body mass 75.4 ± 6.4 kg). Goalkeepers were not included in this investigation. A total of 375 players’ match data were analyzed in this study, as one team has repeated measures. Data were collected from all official home matches played by a single team, along with the corresponding matches of their opponents, using video match analysis. Only players who participated in the entire match (85–95 min) were included in the analysis. Data from players whose playing time fell outside this range were excluded. Specifically, all players who were sent off during the match due to a red card or accumulation of yellow cards, as well as those with limited playing time, were excluded (e.g., substitutions made during the match).

### 2.2. Procedure

Teams were categorized into high (*HR*), medium (*MR*), and low (*LR*) ranking based on their final standing in the Italian championship: 1st–7th (HR), 8th–14th (MR), and 15th–20th (LR). The division of team rankings into three groups of 7, 7, and 6 teams was based on the following considerations: the top 7 teams were selected due to their participation in the Coppa Italia, which could influence their locomotor activity. Additionally, we aimed to analyze the effort of teams competing for the top 7 positions (European competitions or cup qualifications) as well as those fighting relegation. Teams ranked 8th to 14th, with less at stake, provided a balanced contrast between both extremes of the competition spectrum. The playing formations analyzed were 4-4-2, 3-4-3, 4-3-3, and 3-5-2. The absence of certain playing formations was assessed based on the average position of players over 90 min; thus, attacking midfielders in the starting lineup who took on predominantly defensive roles during the match were categorized as part of the midfield, while wide midfielders acting as wingers were classified within the purely offensive area. Comparisons among different team playing formations, both within and across the ranking categories, were conducted.

**Figure 1:**
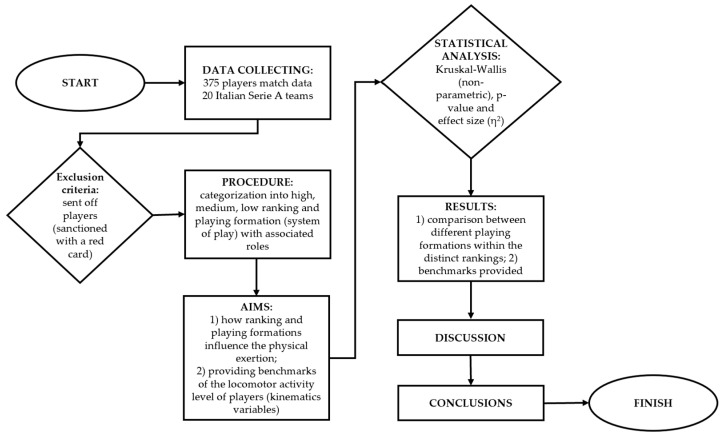
Flow chart of the study design.

For the second aim of the study, the T-score method was employed to provide benchmarks and to facilitate the interpretation of the locomotor activity level of players [26]. The T-score offers a more intuitive alternative to the z-score [27] and is calculated as follows: (Z-score × 10) + 50, with a score of 50 rather than 0, equaling the mean. For enhanced interpretation, these T-score values were combined with qualitative descriptions ranging from “*extremely poor*” (<20) to “*excellent*” (>80). The study design is shown in Figure 1.

The following kinematic variables were analyzed: average metabolic power (*AMP*, w·kg^−1^), average speed (*AS*, m·min^−1^), high metabolic power distance (*HMPD*, >20 w·kg^−1^), very high metabolic power distance (*VHMPD*, >35 w·kg^−1^), high-speed running distance (*HSR*, distance covered above 20 km/h), and, finally, very high-speed running distance (*VHSR*, distance covered at more than 25 km/h). Acceleration events were defined based on Sonderegger’s equation [24] modified by Savoia et al. [28], where an event was considered an acceleration if it exceeded 50% of the a_max_ achievable by the player considering the initial speed. High acceleration data were defined as a percentage of the total acceleration time (*H-acc*). High decelerations were defined as a percentage of the total deceleration time through an absolute threshold (greater than 2 m·s^−2^, *H-dec*).

**Figure 2:**
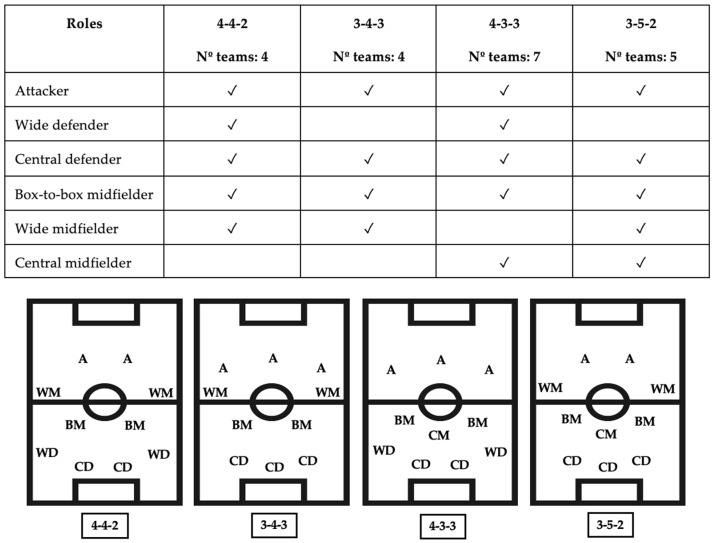
Playing formation and roles.

Missing data or data that did not meet the inclusion criteria were excluded. Subsequently, the players were categorized based on their playing formation and role, as shown in Figure 2.

These experimental procedures were approved by the local Human Ethics Committee of Liverpool John Moores University (No. 12/SPS/003). The study complied with the Declaration of Helsinki.

### 2.3. Video Match Analysis

Match analysis was performed using the validated multi-camera video analysis system Stats Perform’s SportVU (Stats Perform, Chicago, IL, USA), tracking at up to 25 Hz rates. The Technical University of Munich (TUM) determined the measurement accuracy of this device with a typical error of 2.7% for total distance [29]. Raw data were provided via Cartesian coordinates by K-Sport (K-Sport World SRL), and primary data were smoothed at 5 Hz. The Stats SportVU tracking system provides the performance data by extracting and processing the coordinates of players (X, Y) and the ball (X, Y, Z) through HD cameras as well as sophisticated software and statistical algorithms [29]. Player movements were captured during matches through cameras located at the roof level. Data were analyzed using STATS Viewer and K-Sport Dynamix, and were processed through the K-Filter software package (version K1.24, K-Sport World SRL) to create a dataset on each player’s physical and technical performance.

### 2.4. Statistical Analysis

Based on the objectives of the study, the first analysis examined locomotor activity (dependent variable) across different rankings (independent variable), while the second analysis aimed to assess whether locomotor activity (dependent variable) differs among various playing formations (independent variable) within the same ranking zone. A Shapiro–Wilk test was used to test the normal distribution of the data. Not following a normal distribution, a non-parametric statistical analysis was applied to the data. Comparisons between groups were accomplished via the Kruskal–Wallis [30] test, which is a valid non-parametric alternative to one-way ANOVA. It extends the two-sample Wilcoxon test when there are more than two groups to compare. When the *p*-value was <0.05, the Dunn test [31] with Bonferroni adjustment was applied to discriminate which group was different from the others. The Epsilon squared (η^2^) was reported as effect size (ES) according to Tomczak and Tomczak [32]: η^2^ ≤ 0.06 (small effect), 0.06 < η^2^ < 0.14 (moderate effect), and η^2^ ≥ 0.14 (large effect). Significance was accepted at an alpha level of *p* < 0.05. All statistical analyses were performed using R (version 4.1.1) [33] and the package rstatix [34].

## 3. Results

The first comparison was conducted to see if there were any differences among the teams according to their position in the rankings. Results are synthesized in Table 1, Table 2, Table 3 and Table 4.

Small statistical differences (*p* < 0.001) were found among different rankings for the variables H-acc and AS. The Dunn test with Bonferroni adjustment showed that teams in the MR reported a lower H-acc than HR and LR (ES = small), while there were no significant differences between HR and LR. Moreover, HR teams reported lower AS than MR and LR teams (ES = small), with no differences, in this case, between MR and LR.

As shown in Table 1, where differences among playing formations within the same ranking group were assessed, statistical differences were also detected. In the HR group, differences were found for H-acc and H-dec. Specifically, the H-dec 3-4-3 formation yielded lower results compared to the 3-5-2 and 4-3-3 formations with moderate effect size, whereas for H-acc, the 4-3-3 had values higher than those of 3-4-3, 3-5-2, and 4-4-2. No statistical differences were established among the variables in the MR group. Finally, in the LR group, a large significant difference found was for H-acc, where 4-3-3 < 4-4-2 in playing formation. No other statistical differences were detected.

The t-score values combined with qualitative descriptions for each formation and role are reported in Table 5, Table 6, Table 7 and Table 8.

## 4. Discussion

The aim of this study was to investigate the influence of team ranking and playing formation on the locomotor activity of professional soccer players in the Italian First Division. Additionally, the study also aimed to establish benchmarks combined with qualitative descriptors to provide insight into role-specific locomotor activity of players and to help define performance levels as above or below average.

### 4.1. Differences Among Rankings

Only three statistical differences were detected when differently ranked teams were analyzed. HR teams reported more H-acc than the MR teams (ES = small), partially in agreement with Aquino et al. [11], who noted that the high-ranked teams performed more accelerations compared to the bottom-ranked ones. However, in this investigation, accelerations were comparable between low- and high-ranked teams, emphasizing that the technical and tactical aspects that come into play when trying to avoid relegation play a crucial role for lower-ranked teams, significantly impacting their physical effort.

HR teams showed significantly lower average speed during the match compared to MR and LR teams (ES = small). This contrasts with the findings of Aquino et al. [11], who reported that the top-ranked team covered more distance (and thus had higher average speed) than the lower-ranked teams. Our results suggest that average speed may be less critical for match outcomes, and that high-intensity activities are more important to consider [15]. From a practical perspective, this study finds that H-acc are central to soccer performance, fully aligning with contextual variables analyzed through machine learning techniques [35].

### 4.2. Differences Among Playing Formations Within the Same Ranking Level

In the HR group, the 3-4-3 playing formation reported lower H-dec than the 3-5-2 and 4-3-3 formations (ES = moderate). This result is partially supported by Tierney et al. [36], who identified the following decreasing order in terms of differences between playing systems: 3-5-2 > 3-4-3 > 4-3-3 > 4-4-2.

Borghi et al. [37], and Tierney et al. [36] reported that the 3-5-2 formation executed the greatest amount of accelerations. However, our findings showed that the 4-3-3 formation had the highest H-acc values, with greater acceleration compared to the 3-4-3, 3-5-2, and 4-4-2 formations (ES = large). These results are consistent with the findings of Morgans et al. [7] who reported that the 4-3-3 formation resulted in more acceleration than the 3-5-2 formation when comparing teams primarily focused on defending collectively in a deep position (with very low ball possession/low-block). Furthermore, these findings imply that coaches can assess running performance based on formation, allowing them to adjust tactical strategies to enhance physical performance. Nevertheless, our findings were not consistent across all ranking groups, highlighting that the playing formation may influence locomotor activities differently among teams of varying ranks. These differences could be attributed to the way a “flat” midfield defends, with an extra man in the center, given that this role requires expending a lot of energy both in possession and out of possession.

### 4.3. Benchmark of Locomotor Activity

The second purpose of this study was to compile normative data and create benchmarks for AMP, AS, and different high-intensity variables for each role attained by professional soccer players. This approach enables the analysis of players’ kinematic variables, allowing us to understand if their performance is above or below average, as supported by Laterza and Manzi [26]. Moreover, the data collected could be used to assess players’ fatigue and detect symptoms of overreaching or overtraining. If a player consistently exhibits poor performance over a prolonged period, this could be an early sign of overtraining [38]. In addition, these benchmarks may represent a useful tool to assess the performance of junior professionals competing for the first year at a professional level. They can help determine if their level is comparable with more experienced professionals, provide insights into their training needs, and facilitate the monitoring of their performance parameters over time [26].

Analyzing various playing formations and role positions is crucial in soccer, as each distinct role demands a unique activity profile [17,18]. For instance, the average distance covered above 25 km/h by attackers differs among playing formations and roles. A distance that might be considered average in one formation could be subpar in another. To illustrate, an attacker in a 3-5-2 formation might cover 260 m at high speed, which could be significantly less than what is expected for the same role in a 4-3-3 formation (see Table 5, Table 6, Table 7 and Table 8). This analysis provides invaluable insights for coaches, allowing them to tailor training programs to the specific demands of different roles and formations. Furthermore, benchmarks offer additional benefits. By examining the range of performance levels for each role, we can identify positions where performance is more consistent (i.e., a smaller range). This suggests a more clearly defined activity profile for that role. For example, in a 3-4-3 formation, the box-to-box midfielder’s performance might be more consistent than that of a wide midfielder. This research has successfully provided readily available data for professional soccer coaches, enabling them to quickly assess their athletes’ performance levels. Additionally, the data can help identify players with greater work capacity, potentially allowing coaches to assign them specialized tactical roles that leverage their superior abilities without compromising their performance.

Finally, a further practical application that combines normative data, formation, and ranking should be placed in a higher hierarchical context, such as talent selection. Indeed, the discussion on the crucial importance of neuromuscular qualities in soccer does not imply the absence of intense accelerations in daily training (e.g., SSGs) [39], but rather encourages the search for players who genetically respond to specific strength characteristics, as is the case with the metabolic aspect and the multidisciplinary approach to talent identification [40].

### 4.4. Limitations

While this study provides valuable insights, it is important to recognize its inherent limitations, which may influence the interpretation and generalizability of the findings.

This study is based on a sample of matches that is not uniform in terms of home and away games. The game location factor must be considered in the analysis of the players’ physical data, as it represents a critical piece of information. It is directly correlated with the style of play and consequently influences the intensity of the performance, as demonstrated by Hands et al. [41] and Beato et al. [42]. Moreover, other contextual variables, such as ball possession, match results, and playing strategies (e.g., high-press, counterattacks, deep-defending) both from an individual and collective tactical perspective, were not considered, which could also impact the outcomes [43,44,45].

It must be noted that the dynamic nature of the game cannot be analyzed by simplifying match effects through the (static) initial player formation. Instead, such an approach should be implemented through the flow of technical-tactical movements, which characterize a comprehensive framework to assess tactical situations as key performance indicators (KPIs) in soccer [46].

Researchers and practitioners should also be mindful of some aspects of this study before using the presented normative data. The data collected refer to Premier Division Championship (Serie A) players, meaning that professional soccer players competing in other championships (e.g., the Spanish LaLiga and the English FA Premier League) might have different activity profiles, as supported by Dellal et al. [47].

Lastly, to the best of our knowledge, this methodological approach, developed by Sonderegger et al. [24], typically utilizes a spatial reference (distance covered in meters), whereas in this study, the quantitative variable was temporal (the sum of short time intervals as a percentage of the total time spent accelerating during the match). In practice, however, this approach does not consider the total number of accelerations (n°. of events), which can make comparisons with other studies difficult. Following the previous concept, still in terms of time spent, a fixed threshold of 2 m·s^−2^ was used for decelerations. Therefore, readers should be mindful when interpreting our speed variations data (H-acc and H-dec), as any comparison with other studies may require careful consideration.

## 5. Conclusions

This study provides insights into the influence of team ranking and playing formation on the locomotor activities of professional soccer players in the Italian First Division. The results revealed that HR teams exhibit a higher percentage of high-intensity accelerations compared to MR teams, emphasizing the importance of high-intensity efforts over average speed in determining match outcomes. However, these differences varied across rankings, highlighting the variability in physical demands based on team strategy and opposition.

The study also demonstrated that playing formations significantly impact locomotor activities, with the 4-3-3 formation showing greater acceleration demands than others, such as 3-5-2 and 4-4-2. These differences were most pronounced in HR teams, underscoring the strategic role of formation in optimizing player performance. However, the lack of consistent trends across all ranking groups suggests that the effectiveness of a formation may vary depending on the team’s ranking.

Furthermore, the benchmarks and normative data provided for various roles and formations offer valuable tools for coaches to assess player performance and detect signs of overtraining. By understanding the role-specific demands within different formations, coaches can better tailor training programs to enhance player readiness and performance.

In the authors’ opinion, due to the fact that accelerations represent one of the most predictive variables associated with the outcome of the match [35], it was essential to improve the reliability of the acceleration data using the method proposed by Sonderegger et al. [24].

Future research should address the current investigation’s limitations and explore the evolving dynamics of locomotor activities in modern soccer. Nevertheless, the data generated in this study contribute to a better understanding of the physical demands in soccer and provide a foundation for further investigations.

## Figures and Tables

**Table 1:** Kruskal–Wallis and Dunn tests considering ranking as an explicative variable.

	Ranking	VHSR (m)	HSR (m)	HMPD (m)	H-dec (%)	H-acc (%)	AMP (w·kg^−1^)	AS (m·min^−1^)	VHMPD (m)
Kruskal–Wallis *p*-value		>0.05	>0.05	>0.05	>0.05	†	>0.05	†	>0.05
Dunn test adjusted *p*-value	HR-MR	/	/	/	/	†	/	**	/
	HR-LR	/	/	/	/	/	/	†	/
	MR-LR	/	/	/	/	/	/	/	/
Mean Values	HR	308.1	836.7	3081.7	0.14	0.09	11.33	118.9	1080.4
	MR	286.1	779.9	2899.4	0.13	0.08	11.21	123.6	1076.7
	LR	291.9	798.9	3099.4	0.13	0.08	11.59	124.9	1156.9
ES		/	/	/	/	small	/	small	/

AS: average speed; AMP: average metabolic power; ES: effect size; H-acc: % time spent at >50% of max acceleration based on the initial speed; H-dec: % time spent < 2·m^−2^; HMPD: distance covered at w > 20·kg^−1^; HR: high ranking; HSR: distance covered at speed > 20 km/h; LR: low ranking; MR: medium ranking; VHMPD: distance covered at w > 35·kg^−1^; VHSR: distance covered at speed > 25 km/h; **: *p* < 0.01; †: *p* < 0.001.

**Table 2:** Kruskal–Wallis and Dunn tests for high-ranking teams considering playing formations as an explicative variable.

	PF	VHSR (m)	HSR (m)	HMPD (m)	H-dec (%)	H-acc (%)	AMP (w·kg^−1^)	AS (m·min^−1^)	VHMPD (m)
Kruskal–Wallis *p*-value		>0.05	>0.05	>0.05	*	0.000 †	>0.05	>0.05	>0.05
Dunn test adjusted *p*-value	343–352	/	/	/	**	/	/	/	/
	343–433	/	/	/	**	†	/	/	/
	343–442	/	/	/	/	/	/	/	/
	352–433	/	/	/	/	†	/	/	/
	352–442	/	/	/	/	/	/	/	/
	433–442	/	/	/	/	†	/	/	/
Mean Values	343	344.2	841.6	2714.5	0.12	0.07	10.76	120.8	1030.8
	352	271.9	841.2	3150.9	0.14	0.08	11.42	119.7	1061.8
	433	335.3	833.1	2993.6	0.14	0.11	11.25	118.4	1130.8
	442	372.4	787.8	3116.9	0.13	0.07	11.12	106.9	1003.5
ES		/	/	/	moderate	large	/	/	/

AS: average speed; AMP: average metabolic power; ES: effect size; H-acc: % time spent at >50% of max acceleration based on the initial speed; H-dec: % time spent < 2·m^−2^; HMPD: distance covered at w > 20·kg^−1^; HSR: distance covered at speed > 20 km/h; PF: playing formations; VHMPD: distance covered at w > 35·kg^−1^; VHSR: distance covered at speed > 25 km/h; *: *p* < 0.05; **: *p* < 0.01; †: *p* < 0.001.

**Table 3:** Wilcoxon–Mann–Whitney test for medium-ranking teams considering playing formations as an explicative variable.

PF		VHSR (m)	HSR (m)	HMPD (m)	H-dec (%)	H-acc (%)	AMP (w·kg^−1^)	AS (m·min^−1^)	VHMPD (m)
Wilcoxon *p*-value		>0.05	>0.05	>0.05	>0.05	>0.05	>0.05	>0.05	>0.05
Mean Values	343	302.5	783.0	2834.4	0.13	0.08	10.98	121.8	1037.1
	433	311.9	872.1	3025.5	0.13	0.08	11.45	124.5	1149

AS: average speed; AMP: average metabolic power; H-acc: % time spent at >50% of max acceleration based on the initial speed; H-dec: % time spent < 2·m^−2^; HMPD: distance covered at w > 20·kg^−1^; HSR: distance covered at speed > 20 km/h; PF: playing formations; VHMPD: distance covered at w > 35·kg^−1^; VHSR: distance covered at speed > 25 km/h.

**Table 4:** Kruskal–Wallis and Dunn tests for low-ranking teams considering playing formations as an explicative variable.

	PF	VHSR (m)	HSR (m)	HMPD (m)	H-dec (%)	H-acc (%)	AMP (w·kg^−1^)	AS (m·min^−1^)	VHMPD (m)
Kruskal–Wallis *p*-value		>0.05	>0.05	>0.05	>0.05	**	>0.05	>0.05	>0.05
Dunn test adjusted *p*-value	352–433	/	/	/	/	/	/	/	/
	352–442	/	/	/	/	/	/	/	/
	433–442	/	/	/	/	**	/	/	/
Mean Values	352	286.2	855.2	3292.7	0.13	0.08	11.80	130	1210.3
	433	257.2	712.6	3047	0.13	0.07	11.71	115.4	1115.9
	442	308.3	789.5	2980.1	0.14	0.09	11.40	124.6	1133.4
ES		/	/	/	/	large	/	/	/

AS: average speed; AMP: average metabolic power; ES: effect size; H-acc: % time spent at >50% of max acceleration based on the initial speed; H-dec: % time spent < 2·m^−2^; HMPD: distance covered at w > 20·kg^−1^; HSR: distance covered at speed > 20 km/h; PF: playing formations; VHMPD: distance covered at w > 35·kg^−1^; VHSR: distance covered at speed > 25 km/h; **: *p* < 0.01.

**Table 5:** T-scores for the 4-4-2 formation.

Role	T-Score Value	VHSR (m)	HSR (m)	HMPD (m)	H-dec (%)	H-acc (%)	AMP (w·kg^−1^)	AS (m·min^−1^)	VHMPD (m)
	>80	>662	>1496	>4369	>0.2	>0.15	>13.4	>142	>1876
	70–80	544–662	1256–1496	3866–4369	0.18–0.2	0.13–0.15	12.6–13.4	135–142	1607–1876
	60–70	425–544	1015–1256	3363–3866	0.15–0.18	0.11–0.13	11.8–12.6	128–135	1337–1607
	55–60	366–425	895–1015	3111–3363	0.14–0.15	0.1–0.11	11.4–11.8	125–128	1202–1337
Wide Def	45–55	248–366	654–895	2609–3111	0.12–0.14	0.07–0.1	10.6–11.4	118–125	933–1202
(WD)	40–45	189–248	534–654	2357–2609	0.11–0.12	0.06–0.07	10.2–10.6	115–118	798–933
	30–40	70–189	293–534	1854–2357	0.09–0.11	0.04–0.06	9.4–10.2	108–115	528–798
	20–30	0–70	53–293	1351–1854	0.06–0.09	0.02–0.04	8.6–9.4	101–108	259–528
	<20	negative	<53	<1351	<0.06	<0.02	<8.6	<101	<259
	>80	>234	>641	>3159	>0.14	>0.1	>11.7	>128	>1067
	70–80	206–234	568–641	2874–3159	0.13–0.14	0.09–0.1	11.1–11.7	123–128	961–1067
	60–70	178–206	495–568	2588–2874	0.13–0.13	0.08–0.09	10.6–11.1	118–123	856–961
	55–60	164–178	458–495	2446–2588	0.12–0.13	0.08–0.08	10.4–10.6	116–118	803–856
Cent Def	45–55	136–164	385–458	2161–2446	0.11–0.12	0.07–0.08	9.9–10.4	111–116	698–803
(CD)	40–45	122–136	349–385	2018–2161	0.11–0.11	0.06–0.07	9.6–9.9	108–111	645–698
	30–40	93–122	276–349	1733–2018	0.1–0.11	0.05–0.06	9.1–9.6	103–108	539–645
	20–30	65–93	203–276	1448–1733	0.09–0.1	0.04–0.05	8.6–9.1	98–103	434–539
	<20	<65	<203	<1448	<0.09	<0.04	<8.6	<98	<434
	>80	>324	>1029	>4649	>0.21	>0.15	>14.7	>162	>1998
	70–80	280–324	927–1029	4217–4649	0.19–0.21	0.14–0.15	13.9–14.7	151–162	1749–1998
	60–70	236–280	824–927	3785–4217	0.17–0.19	0.12–0.14	13–13.9	141–151	1500–1749
	55–60	215–236	773–824	3569–3785	0.16–0.17	0.11–0.12	12.6–13	135–141	1376–1500
Btob Mid	45–55	171–215	670–773	3137–3569	0.14–0.16	0.09–0.11	11.8–12.6	124–135	1127–1376
(BM)	40–45	149–171	619–670	2921–3137	0.13–0.14	0.08–0.09	11.3–11.8	119–124	1003–1127
	30–40	106–149	516–619	2489–2921	0.11–0.13	0.06–0.08	10.5–11.3	108–119	754–1003
	20–30	62–106	414–516	2057–2489	0.09–0.11	0.04–0.06	9.6–10.5	97–108	505–754
	<20	<62	<414	<2057	<0.09	<0.04	<9.6	<97	<505
	>80	>771	>1768	>5446	>0.21	>0.13	>14.8	>148	>1896
	70–80	655–771	1539–1768	4802–5446	0.18–0.21	0.12–0.13	13.9–14.8	142–148	1696–1896
	60–70	539–655	1310–1539	4158–4802	0.16–0.18	0.1–0.12	12.9–13.9	136–142	1496–1696
	55–60	481–539	1195–1310	3836–4158	0.15–0.16	0.1–0.1	12.5–12.9	133–136	1396–1496
Wide Mid	45–55	365–481	966–1195	3192–3836	0.13–0.15	0.09–0.1	11.5–12.5	127–133	1196–1396
(WM)	40–45	307–365	852–966	2870–3192	0.12–0.13	0.08–0.09	11–11.5	124–127	1096–1196
	30–40	190–307	623–852	2226–2870	0.1–0.12	0.07–0.08	10.1–11	117–124	896–1096
	20–30	74–190	394–623	1582–2226	0.07–0.1	0.06–0.07	9.1–10.1	111–117	696–896
	<20	<74	<394	<1582	<0.07	<0.06	<9.1	<111	<696
	>80	>965	>1764	>4776	>0.21	>0.17	>14.9	>158	>1881
	70–80	773–965	1493–1764	4230–4776	0.19–0.21	0.14–0.17	13.8–14.9	147–158	1651–1881
	60–70	580–773	1221–1493	3683–4230	0.17–0.19	0.12–0.14	12.7–13.8	136–147	1422–1651
	55–60	484–580	1085–1221	3410–3683	0.16–0.17	0.11–0.12	12.2–12.7	130–136	1307–1422
Attackers	45–55	291–484	814–1085	2864–3410	0.13–0.16	0.08–0.11	11.1–12.2	119–130	1078–1307
(A)	40–45	195–291	678–814	2590–2864	0.12–0.13	0.07–0.08	10.5–11.1	114–119	963–1078
	30–40	2–195	407–678	2044–2590	0.1–0.12	0.05–0.07	9.4–10.5	103–114	734–963
	20–30	0–2	135–407	1498–2044	0.08–0.1	0.03–0.05	8.3–9.4	92–103	505–734
	<20	negative	<135	<1498	<0.08	<0.03	<8.3	<92	<505

AS: average speed; AMP: average metabolic power; H-acc: % time spent at >50% of max acceleration based on the initial speed; H-dec: % time spent < 2·m^−2^; HMPD: distance covered at w > 20·kg^−1^; HSR: distance covered at speed > 20 km/h; T-score: >80 (*excellent*), 70–80 (*very good*), 60–70 (*good*), 55–60 (*above average*), 45–55 (*average*), 40–45 (*below average*), 30–40 (*poor*), 20–30 (*very poor*), <20 (*extremely poor*); VHMPD: distance covered at w > 35·kg^−1^; VHSR: distance covered at speed > 25 km/h.

**Table 6:** T-scores for the 4-3-3 formation.

Role	T-Score Value	VHSR (m)	HSR (m)	HMPD (m)	H-dec (%)	H-acc (%)	AMP (w·kg^−1^)	AS (m·min^−1^)	VHMPD (m)
	>80	>625	>1633	>4348	>0.17	>0.16	>13.4	>141	>1712
	70–80	531–625	1397–1633	3925–4348	0.16–0.17	0.14–0.16	12.7–13.4	135–141	1529–1712
	60–70	438–531	1160–1397	3503–3925	0.15–0.16	0.12–0.14	12.1–12.7	128–135	1345–1529
	55–60	391–438	1041–1160	3291–3503	0.14–0.15	0.11–0.12	11.7–12.1	125–128	1254–1345
Wide Def	45–55	298–391	805–1041	2868–3291	0.13–0.14	0.09–0.11	11.1–11.7	119–125	1070–1254
(WD)	40–45	251–298	686–805	2657–2868	0.12–0.13	0.08–0.09	10.8–11.1	116–119	979–1070
	30–40	157–251	450–686	2234–2657	0.11–0.12	0.06–0.08	10.1–10.8	109–116	795–979
	20–30	64–157	213–450	1811–2234	0.1–0.11	0.04–0.06	9.4–10.1	103–109	612–795
	<20	<64	<213	<1811	<0.1	<0.04	<9.4	<103	<612
	>80	>440	>1107	>3840	>0.18	>0.14	>13	>132	>1432
	70–80	356–440	919–1107	3354–3840	0.16–0.18	0.12–0.14	12.1–13	125–132	1238–1432
	60–70	272–356	731–919	2868–3354	0.14–0.16	0.1–0.12	11.2–12.1	117–125	1045–1238
	55–60	231–272	638–731	2625–2868	0.13–0.14	0.09–0.1	10.7–11.2	113–117	948–1045
Cent Def	45–55	147–231	450–638	2139–2625	0.11–0.13	0.08–0.09	9.8–10.7	106–113	754–948
(CD)	40–45	105–147	356–450	1896–2139	0.1–0.11	0.07–0.08	9.3–9.8	102–106	658–754
	30–40	21–105	169–356	1410–1896	0.08–0.1	0.05–0.07	8.4–9.3	95–102	464–658
	20–30	0–21	0–169	924–1410	0.07–0.08	0.03–0.05	7.4–8.4	87–95	270–464
	<20	negative	negative	<924	<0.07	<0.03	<7.4	<87	<270
	>80	>551	>1573	>4936	>0.21	>0.17	>14.9	>203	>1919
	70–80	468–551	1371–1573	4504–4936	0.19–0.21	0.15–0.17	14.1–14.9	177–203	1741–1919
	60–70	384–468	1169–1371	4073–4504	0.17–0.19	0.13–0.15	13.3–14.1	151–177	1563–1741
	55–60	343–384	1067–1169	3857–4073	0.16–0.17	0.12–0.13	12.9–13.3	138–151	1474–1563
Btob Mid	45–55	259–343	865–1067	3425–3857	0.14–0.16	0.1–0.12	12.1–12.9	113–138	1295–1474
(BM)	40–45	217–259	764–865	3210–3425	0.13–0.14	0.09–0.1	11.7–12.1	100–113	1206–1295
	30–40	134–217	561–764	2778–3210	0.11–0.13	0.06–0.09	10.9–11.7	74–100	1028–1206
	20–30	51–134	359–561	2347–2778	0.09–0.11	0.04–0.06	10.1–10.9	48–74	850–1028
	<20	<51	<359	<2347	<0.09	<0.04	<10.1	<48	<850
	>80	>325	>874	>4143	>0.18	>0.13	>14.2	>157	>1474
	70–80	272–325	769–874	3734–4143	0.16–0.18	0.12–0.13	13.2–14.2	145–157	1326–1474
	60–70	218–272	664–769	3325–3734	0.15–0.16	0.1–0.12	12.3–13.2	133–145	1179–1326
	55–60	192–218	611–664	3120–3325	0.14–0.15	0.1–0.1	11.8–12.3	127–133	1106–1179
Cent Mid	45–55	138–192	505–611	2712–3120	0.13–0.14	0.08–0.1	10.9–11.8	115–127	958–1106
(CM)	40–45	112–138	453–505	2507–2712	0.12–0.13	0.07–0.08	10.4–10.9	109–115	885–958
	30–40	58–112	347–453	2098–2507	0.11–0.12	0.06–0.07	9.4–10.4	97–109	738–885
	20–30	5–58	242–347	1689–2098	0.09–0.11	0.04–0.06	8.5–9.4	85–97	590–738
	<20	<5	<242	<1689	<0.09	<0.04	<8.5	<85	<590
	>80	>748	>1615	>4444	>0.2	>0.16	>14.4	>148	>1779
	70–80	629–748	1400–1615	3962–4444	0.18–0.2	0.14–0.16	13.3–14.4	139–148	1578–1779
	60–70	511–629	1185–1400	3480–3962	0.16–0.18	0.12–0.14	12.3–13.3	129–139	1378–1578
	55–60	452–511	1078–1185	3239–3480	0.15–0.16	0.11–0.12	11.8–12.3	125–129	1277–1378
Attackers	45–55	333–452	863–1078	2756–3239	0.13–0.15	0.09–0.11	10.7–11.8	115–125	1077–1277
(A)	40–45	274–333	755–863	2515–2756	0.12–0.13	0.08–0.09	10.2–10.7	110–115	976–1077
	30–40	155–274	541–755	2033–2515	0.1–0.12	0.06–0.08	9.2–10.2	101–110	776–976
	20–30	37–155	326–541	1551–2033	0.08–0.1	0.04–0.06	8.2–9.2	91–101	575–776
	<20	<37	<326	<1551	<0.08	<0.04	<8.2	<91	<575

AS: average speed; AMP: average metabolic power; H-acc: % time spent at >50% of max acceleration based on the initial speed; H-dec: % time spent < 2·m^−2^; HMPD: distance covered at w > 20·kg^−1^; HSR: distance covered at speed > 20 km/h; T-score: >80 (*excellent*), 70–80 (*very good*), 60–70 (*good*), 55–60 (*above average*), 45–55 (*average*), 40–45 (*below average*), 30–40 (*poor*), 20–30 (*very poor*), <20 (*extremely poor*); VHMPD: distance covered at w > 35·kg^−1^; VHSR: distance covered at speed > 25 km/h.

**Table 7:** T-scores for the 3-5-2 formation.

Role	T-Score Value	VHSR (m)	HSR (m)	HMPD (m)	H-dec (%)	H-acc (%)	AMP (w·kg^−1^)	AS (m·min^−1^)	VHMPD (m)
	>80	>482	>1107	>4046	>0.19	>0.13	>13.5	>142	>1370
	70–80	399–482	946–1107	3578–4046	0.17–0.19	0.11–0.13	12.5–13.5	132–142	1200–1370
	60–70	315–399	784–946	3110–3578	0.15–0.17	0.1–0.11	11.6–12.5	122–132	1031–1200
	55–60	273–315	703–784	2875–3110	0.14–0.15	0.09–0.1	11.1–11.6	118–122	946–1031
Cent Def	45–55	190–273	542–703	2407–2875	0.12–0.14	0.07–0.09	10.1–11.1	108–118	777–946
(CD)	40–45	148–190	461–542	2173–2407	0.11–0.12	0.06–0.07	9.6–10.1	103–108	692–777
	30–40	64–148	300–461	1705–2173	0.09–0.11	0.04–0.06	8.7–9.6	93–103	523–692
	20–30	0–64	138–300	1236–1705	0.07–0.09	0.02–0.04	7.7–8.7	83–93	353–523
	<20	negative	< 138	<1236	<0.07	<0.02	<7.7	<83	<353
	>80	>653	>1670	>5292	>0.2	>0.16	>15	>159	>1982
	70–80	540–653	1434–1670	4779–5292	0.19–0.2	0.14–0.16	14.2–15	149–159	1748–1982
	60–70	428–540	1197–1434	4266–4779	0.17–0.19	0.11–0.14	13.3–14.2	140–149	1515–1748
	55–60	372–428	1079–1197	4009–4266	0.16–0.17	0.1–0.11	12.9–13.3	135–140	1398–1515
Btob Mid	45–55	260–372	842–1079	3496–4009	0.14–0.16	0.08–0.1	12–12.9	125–135	1165–1398
(BM)	40–45	203–260	724–842	3240–3496	0.13–0.14	0.07–0.08	11.6–12	120–125	1048–1165
	30–40	91–203	487–724	2726–3240	0.11–0.13	0.05–0.07	10.7–11.6	111–120	814–1048
	20–30	0–91	251–487	2213–2726	0.1–0.11	0.02–0.05	9.8–10.7	101–111	581–814
	<20	negative	<251	<2213	<0.1	<0.02	<9.8	< 101	<581
	>80	>874	>1824	>4915	>0.19	>0.14	>13.7	>146	>1768
	70–80	749–874	1609–1824	4451–4915	0.17–0.19	0.13–0.14	13.1–13.7	138–146	1596–1768
	60–70	624–749	1393–1609	3986–4451	0.16–0.17	0.11–0.13	12.4–13.1	131–138	1424–1596
	55–60	562–624	1285–1393	3754–3986	0.15–0.16	0.1–0.11	12.1–12.4	128–131	1338–1424
Wide Mid	45–55	437–562	1070–1285	3290–3754	0.13–0.15	0.08–0.1	11.4–12.1	120–128	1166–1338
(WM)	40–45	375–437	962–1070	3057–3290	0.12–0.13	0.07–0.08	11.1–11.4	117–120	1080–1166
	30–40	250–375	747–962	2593–3057	0.11–0.12	0.06–0.07	10.5–11.1	110–117	908–1080
	20–30	126–250	531–747	2129–2593	0.09–0.11	0.04–0.06	9.8–10.5	103–110	736–908
	<20	<126	<531	<2129	<0.09	<0.04	<9.8	<103	<736
	>80	>439	>1170	>4745	>0.17	>0.13	>14	>157	>1843
	70–80	349–439	969–1170	4190–4745	0.16–0.17	0.11–0.13	13–14	145–157	1560–1843
	60–70	259–349	768–969	3636–4190	0.15–0.16	0.1–0.11	13–13	134–145	1278–1560
	55–60	214–259	667–768	3358–3636	0.14–0.15	0.09–0.1	12–13	128–134	1137–1278
Cent Mid	45–55	125–214	466–667	2804–3358	0.13–0.14	0.07–0.09	11–12	116–128	854–1137
(CM)	40–45	80–125	365–466	2526–2804	0.13–0.13	0.06–0.07	11–11	111–116	713–854
	30–40	0–80	164–365	1972–2526	0.11–0.13	0.04–0.06	10–11	99–111	430–713
	20–30	negative	0–164	1417–1972	0.1–0.11	0.03–0.04	9–10	88–99	147–430
	<20	negative	negative	<1417	<0.1	<0.03	<9	<88	<147
	>80	>611	>1482	>4530	>0.19	>0.14	>13.8	>144	>1635
	70–80	507–611	1272–1482	4035–4530	0.17–0.19	0.12–0.14	13–13.8	136–144	1446–1635
	60–70	403–507	1062–1272	3541–4035	0.15–0.17	0.1–0.12	12.2–13	128–136	1256–1446
	55–60	351–403	957–1062	3294–3541	0.14–0.15	0.09–0.1	11.8–12.2	124–128	1162–1256
Attackers	45–55	247–351	747–957	2799–3294	0.13–0.14	0.07–0.09	11–11.8	116–124	972–1162
(A)	40–45	194–247	642–747	2552–2799	0.12–0.13	0.06–0.07	10.6–11	112–116	878–972
	30–40	90–194	432–642	2057–2552	0.1–0.12	0.05–0.06	9.8–10.6	105–112	688–878
	20–30	0–90	222–432	1563–2057	0.08–0.1	0.03–0.05	9–9.8	97–105	499–688
	<20	negative	<222	<1563	<0.08	<0.03	<9	<97	<499

AS: average speed; AMP: average metabolic power; H-acc: % time spent at >50% of max acceleration based on the initial speed; H-dec: % time spent < 2·m^−2^; HMPD: distance covered at w > 20·kg^−1^; HSR: distance covered at speed > 20 km/h; T-score: >80 (*excellent*), 70–80 (*very good*), 60–70 (*good*), 55–60 (*above average*), 45–55 (*average*), 40–45 (*below average*), 30–40 (*poor*), 20–30 (*very poor*), <20 (*extremely poor*); VHMPD: distance covered at w > 35·kg^−1^; VHSR: distance covered at speed > 25 km/h.

**Table 8:** T-scores for the 3-4-3 formation.

Role	T-Score Value	VHSR (m)	HSR (m)	HMPD (m)	H-dec (%)	H-acc (%)	AMP (w·kg^−1^)	AS (m·min^−1^)	VHMPD (m)
	>80	>478	>1044	>3449	>0.18	>0.11	>12.9	>139	>1369
	70–80	399–478	903–1044	3106–3449	0.16–0.18	0.1–0.11	12.1–12.9	131–139	1210–1369
	60–70	321–399	762–903	2764–3106	0.14–0.16	0.09–0.1	11.2–12.1	123–131	1052–1210
	55–60	282–321	692–762	2592–2764	0.14–0.14	0.08–0.09	10.8–11.2	119–123	972–1052
Cent Def	45–55	203–282	551–692	2249–2592	0.12–0.14	0.07–0.08	10–10.8	111–119	813–972
(CD)	40–45	164–203	481–551	2078–2249	0.11–0.12	0.06–0.07	9.5–10	107–111	734–813
	30–40	86–164	340–481	1735–2078	0.09–0.11	0.05–0.06	8.7–9.5	99–107	575–734
	20–30	8–86	199–340	1392–1735	0.08–0.09	0.04–0.05	7.9–8.7	91–99	417–575
	<20	<8	<199	<1392	<0.08	<0.04	<7.9	<91	<417
	>80	>492	>1374	>4211	>0.18	>0.13	>14	>151	>1727
	70–80	420–492	1196–1374	3875–4211	0.16–0.18	0.11–0.13	13.2–14	143–151	1545–1727
	60–70	348–420	1017–1196	3538–3875	0.15–0.16	0.1–0.11	12.4–13.2	136–143	1364–1545
	55–60	312–348	928–1017	3369–3538	0.14–0.15	0.09–0.1	12.1–12.4	133–136	1273–1364
Btob Mid	45–55	239–312	750–928	3032–3369	0.13–0.14	0.07–0.09	11.3–12.1	125–133	1091–1273
(BM)	40–45	203–239	661–750	2864–3032	0.12–0.13	0.07–0.07	10.9–11.3	122–125	1001–1091
	30–40	131–203	482–661	2527–2864	0.11–0.12	0.05–0.07	10.2–10.9	115–122	819–1001
	20–30	58–131	304–482	2190–2527	0.09–0.11	0.03–0.05	9.4–10.2	107–115	638–819
	<20	<58	<304	<2190	<0.09	<0.03	<9.4	<107	<638
	>80	>815	>1558	>4529	>0.2	>0.12	>14.1	>151	>1640
	70–80	673–815	1341–1558	4018–4529	0.18–0.2	0.11–0.12	13.2–14.1	142–151	1458–1640
	60–70	530–673	1123–1341	3507–4018	0.15–0.18	0.1–0.11	12.2–13.2	133–142	1276–1458
	55–60	459–530	1014–1123	3252–3507	0.14–0.15	0.09–0.1	11.7–12.2	129–133	1185–1276
Wide Mid	45–55	316–459	797–1014	2741–3252	0.12–0.14	0.07–0.09	10.7–11.7	120–129	1003–1185
(WM)	40–45	245–316	688–797	2485–2741	0.11–0.12	0.07–0.07	10.2–10.7	116–120	912–1003
	30–40	102–245	470–688	1974–2485	0.09–0.11	0.05–0.07	9.3–10.2	107–116	731–912
	20–30	0–102	252–470	1464–1974	0.06–0.09	0.04–0.05	8.3–9.3	99–107	549–731
	<20	negative	<252	<1464	<0.06	<0.04	<8.3	<99	<549
	>80	>841	>1708	>4546	>0.18	>0.13	>14.5	>160	>1744
	70–80	680–841	1430–1708	3963–4546	0.16–0.18	0.11–0.13	13.3–14.5	147–160	1510–1744
	60–70	518–680	1152–1430	3379–3963	0.14–0.16	0.1–0.11	12–13.3	134–147	1276–1510
	55–60	438–518	1013–1152	3088–3379	0.13–0.14	0.09–0.1	11.4–12	127–134	1159–1276
Attackers	45–55	276–438	736–1013	2504–3088	0.11–0.13	0.07–0.09	10.1–11.4	114–127	924–1159
(A)	40–45	195–276	597–736	2213–2504	0.1–0.11	0.06–0.07	9.5–10.1	108–114	807–924
	30–40	34–195	319–597	1629–2213	0.08–0.1	0.05–0.06	8.2–9.5	95–108	573–807
	20–30	0–34	41–319	1046–1629	0.06–0.08	0.03–0.05	7–8.2	81–95	339–573
	<20	negative	<41	<1046	<0.06	<0.03	<7	<81	<339

AS: average speed; AMP: average metabolic power; H-acc: % time spent at >50% of max acceleration based on the initial speed; H-dec: % time spent < 2 m^−2^; HMPD: distance covered at w > 20 kg^−1^; HSR: distance covered at speed > 20 km/h; T-score: >80 (*excellent*), 70–80 (*very good*), 60–70 (*good*), 55–60 (*above average*), 45–55 (*average*), 40–45 (*below average*), 30–40 (*poor*), 20–30 (*very poor*), <20 (*extremely poor*); VHMP: distance covered at w > 35 kg^−1^; VHSR: distance covered at speed > 25 km/h.

## Data Availability

Data are available upon request from the corresponding authors due to privacy and ethical restrictions.
